# Carbapenem non-susceptibility of *Klebsiella pneumoniae* isolates in hospitals from 2011 to 2016, data from the German Antimicrobial Resistance Surveillance (ARS)

**DOI:** 10.1186/s13756-018-0362-9

**Published:** 2018-06-05

**Authors:** Uwe Koppe, Anja von Laer, Lars E. Kroll, Ines Noll, Marcel Feig, Marc Schneider, Hermann Claus, Tim Eckmanns, Muna Abu Sin

**Affiliations:** 1 Department of Infectious Disease Epidemiology, Robert Koch Institute, Seestr. 10, 13353 Berlin, Germany; 2 Department of Epidemiology and Health Monitoring, Robert Koch Institute, General-Pape-Strasse 62-66, 12101 Berlin, Germany; 3 Postgraduate Training for Applied Epidemiology, Department of Infectious Disease Epidemiology, Robert Koch Institute, Seestr. 10, 13353 Berlin, Germany

**Keywords:** *Klebsiella pneumoniae*, Carbapenem, Surveillance, Antimicrobial, Resistance

## Abstract

**Background:**

Carbapenem resistance in *Klebsiella pneumoniae* is of significant public health concern and recently spread across several countries. We investigated the extent of carbapenem non-susceptibility in *K. pneumoniae* isolates in Germany.

**Methods:**

We analysed 2011–2016 data from the German Antimicrobial Resistance Surveillance (ARS) System, which contains routine data of antimicrobial susceptibility testing from voluntarily participating German laboratories. *Klebsiella pneumoniae* isolates tested resistant or intermediate against an antibiotic were classified as non-susceptible.

**Results:**

We included 154,734 isolates from 655 hospitals in the analysis. Carbapenem non-susceptibility in *K. pneumoniae* isolates was low in Germany 0.63% (95% CI 0.51–0.76%). However, in continuously participating hospitals the number of *K. pneumoniae* isolates almost doubled and we found evidence for a slowly increasing trend for non-susceptibility (OR = 1.20 per year, 95% CI 1.09–1.33, *p* < 0.001). Carbapenem non-susceptibility was highest among isolates from patients aged 20–39 in men but not in women. Moreover, carbapenem non-susceptibility was more frequently reported for isolates from tertiary care, specialist care, and prevention and rehabilitation care hospitals as well as from intensive care units. Co-resistance of carbapenem non-susceptible isolates against antibiotics such as tigecycline, gentamicin, and co-trimoxazole was common. Co-resistance against colistin was 13.3% (95% CI 9.8–17.9%) in carbapenem non-susceptible isolates.

**Conclusion:**

Carbapenem non-susceptibility in *K. pneumoniae* isolates in Germany is still low. However, it is slowly increasing and in the light of the strong increase of *K. pneumoniae* isolates over the last year this poses a significant challenge to public health. Continued surveillance to closely monitor trends as well as infection control and antibiotic stewardship activities are necessary to preserve treatment options.

**Electronic supplementary material:**

The online version of this article (10.1186/s13756-018-0362-9) contains supplementary material, which is available to authorized users.

## Background

*Klebsiella (K.) pneumoniae* is a gram negative pathogen in the family of *Enterobacteriaceae*. It is able to acquire a wide array of antimicrobial resistance (AMR) genes and can cause severe healthcare- associated infections [1, 2]. Infections with carbapenem non-susceptible *K. pneumoniae* are associated with higher mortality and the World Health Organization classified carbapenem-resistant *K. pneumoniae* as a critical priority pathogen for research and development [3, 4]. Carbapenem resistance has been emerging world-wide over the last years with local differences in frequency and mechanisms of resistance [1]. The prevalence of carbapenem non-susceptible *K. pneumoniae* strongly increased in countries of Southern Europe such as Greece and Italy [5]. Germany has experienced local outbreaks of carbapenem-resistant *K. pneumoniae* and other resistant *Enterobacteriaceae* [6, 7]. The German National Reference Centre for multidrug-resistant gram-negative bacteria analyses carbapenem non-susceptible *K. pneumoniae* strains that are submitted for further analysis and confirmation. In a recent analysis, carbapenemases could be identified in 50,9% of carbapenem-resistant *K. pneumoniae* isolates with OXA-48 being the most prevalent [8]. In a recent study in a German academic tertiary care centre, OXA-48 was detected in 19 of 28 carbapenem-resistant *K. pneumoniae* isolates [9].

A pivotal element in the prevention and control of carbapenem-resistant *K. pneumoniae* is the regular and ongoing surveillance of colonizations and infections in order to inform infection control measures in hospitals [10].

The Antimicrobial Resistance Surveillance (ARS) is the national surveillance system for AMR in Germany [11]. Since 2008, microbiological laboratories across Germany voluntarily participate and submit data from routine antimicrobial susceptibility testing to ARS. AMR data for selected pathogens are accessible on the ARS website (https://ars.rki.de). In addition, the surveillance system contributes data to the European Antimicrobial Resistance Surveillance Network (EARS-Net) and to the Global Antimicrobial Resistance Surveillance System (GLASS) at the World Health Organization [5].

In 2016, a national statutory surveillance system for carbapenem non-susceptibility was implemented in Germany. However, information on changes in carbapenem resistance over the recent years is scarce. The objective of our study was to analyse trends and risk-factors for carbapenem non-susceptibility of *K. pneumoniae* isolates in Germany as well as co-resistance of carbapenem non-susceptible isolates to other commonly used antibiotics.

## Methods

### Study design and data source

We undertook an observational cross-sectional study to analyse carbapenem non-susceptibility of *K. pneumoniae* isolates in hospitals in Germany.

Data on antimicrobial susceptibility testing are obtained from ARS. Participating laboratories share their data on routine antimicrobial susceptibility testing (AST) of microbiological samples from hospitals and medical practices [11]. Participation is voluntary for the laboratories and can change over time. The sample of hospitals and medical practices providing data to ARS is organised by laboratory clusters. The geographical distribution of the hospitals contributing data to the study is shown in Additional file 1: Appendix S2. The identity of the hospitals and medical practices is kept confidential. Data on patients is anonymised.

The laboratories identify bacteria from specimens sent in from hospitals or medical practices and determine the zone diameters or minimum inhibitory concentrations (MIC) of routinely used antibiotics (e.g. with microdilution, gradient or disk diffusion). Based on international guidelines (e.g. by the European Committee on Antimicrobial Susceptibility Testing (EUCAST)), the zone diameter or MIC are used for the interpretative results “susceptible” (S), “intermediate” (I), or “resistant” (R) against the tested antibiotic.

All participating laboratories are accredited to perform pathogen identification and antimicrobial susceptibility testing. Data are checked for plausibility during the data transmission process and are validated by the laboratories annually for completeness and consistency.

### Outcomes and covariates

The main outcome of the study is the proportion of carbapenem non-susceptible *K. pneumoniae* isolates in relation to all *K. pneumoniae* isolates tested for carbapenem resistance. An isolate is considered non-susceptible against an antibiotic if the susceptibility test results are interpreted as “resistant” (R) or “intermediate” (I). Age was converted into a categorical variable for the analysis (< 1, 1–19, 20–39, 40–49, 50–59, 60–69, 70–79, and 80+ years). The specimen types were grouped as follows: swabs (swabs from eye, nose, throat, ear, tongue, and urogenital sites as well as intraoperative swabs and other/unspecified swabs), blood (blood cultures), puncture (tissue biopsy, cerebrospinal fluid, and aspirate from pleural cavity, abscess, ascites, or joint puncture, other punctures), urine (urine samples), wound (swabs from wounds and abscesses), respiratory (bronchial lavage, bronchial secretions, sputum, tracheal secretion, other respiratory samples), other (dialysate, ejaculate, catheters, other). To analyse for seasonality, a categorical variable was created according to the month in which the isolate was obtained: January – March, April – June, July – September, October – December. The geographic regions were grouped as follows: Northwest (Bremen, Hamburg, Lower Saxony, Schleswig-Holstein), West (North Rhine-Westphalia), Southwest (Baden-Wuerttemberg, Hesse, Rhineland-Palatinate, Saarland), Southeast (Bavaria, Saxony, Thuringia), and Northeast (Berlin, Brandenburg, Mecklenburg-West Pomerania, Saxony-Anhalt). Several variables on the county level of the hospital were also included in the analysis: counties were divided into “rural” or “city” based on a list from the Federal Agency for Cartography and Geodesy [12, 13]. Moreover, the social deprivation index per county was derived from the German Index of Socioeconomic Deprivation (GISD) [14, 15]. The GISD uses nine indicators from publicly available administrative datasets. It is based on factor analysis for indexing and weighting of the indicators to the three latent dimensions education, occupation and income. For the analysis, a categorical variable was created dividing the counties by social deprivation index into quintiles with 1 indicating the lowest deprivation and 5 the highest. In addition, the density of hospital beds per 10.000 inhabitants was also included on a county level. For the analysis a categorical variable was created dividing the counties by hospital bed densities into quartiles.

Analysed risk factors include age and sex of the patient, hospital care level (Secondary Care, Tertiary Care, Specialist Care, Prevention and Rehabilitation Care, other), type of care (intensive, normal hospital ward, other), clinical speciality (surgery and related, internal medicine, other), specimen type, region, county type (rural, city), social deprivation index of the county where the hospital is located, hospital beds per 10.000 inhabitants in the county where the hospital is located as well as year and quarter when the isolate was obtained.

### In- and exclusion criteria of *K. pneumoniae* isolates

In the analysis, we focused on materials that are most likely derived from clinical samples, so isolates labelled as screening samples, anal swabs, and stool samples were excluded. We excluded isolates without susceptibility testing. In order to avoid bias from repeated testing, we only included the first isolate per patient per quarter in the analysis irrespective of the specimen type. If several isolates from one patient were tested on the same day we selected the most relevant isolate for the analysis according to this priority: isolate tested non-susceptible against at least one carbapenem > isolate tested against at least one carbapenem > isolate not tested against at least one carbapenem.

### Statistical analysis

The distribution of baseline characteristics of the *K. pneumoniae* isolates was analysed using percentages and 95% confidence intervals (95% CI) for categorical variables accounting for clustering on the hospital level. Continuous variables were analysed as means with standard deviations if normally distributed and as median with interquartile ranges if non-normally distributed.

Carbapenem non-susceptibility was defined as the proportion of non-susceptible isolates in relation to all tested isolates in the analysis and expressed in percentage and 95% confidence intervals accounting for clustering on the hospital level. An isolate was considered non-susceptible to at least one carbapenem if it was tested as intermediate or resistant against meropenem, imipenem, or ertapenem.

Risk factors for carbapenem non-susceptibility were analysed using univariable and multivariable multilevel (hierarchical) mixed-effects logistic regression models with random intercepts, accounting for clustering on the county and hospital level. Mixed models allow calculating intraclass correlation coefficients for the random intercepts, to quantify variance on different levels [16]. *P*-values were calculated using Wald tests. For the multivariable model, year, quarter, age and sex of patient, hospital care level, type of care, clinical speciality, specimen type, region, county type, county deprivation index, and hospital beds per 10,000 inhabitants were included. Isolates with missing data in any of these variables were not included in the uni- and multivariable regression models (full case analysis).

For the analysis of carbapenem non-susceptibility over time, a univariable multilevel (hierarchical) mixed-effects logistic regression model was calculated with year as a continuous variable. Only isolates from hospitals that continuously contributed data from 2011 to 2016 were included in the analysis. The *p*-value was derived from a Wald test.

### Sensitivity analyses

Not all isolates have been tested for resistance against all three carbapenems (meropenem, imipenem, and ertapenem) and we conducted a sensitivity analysis restricted to isolates that were tested against all three carbapenems.

Differences in EUCAST and CLSI breakpoints might lead to different interpretations of carbapenem non-susceptibility. Consequently, in some cases an isolate might have been categorized as “sensitive” according to one standard and as “intermediate” (i.e. non-susceptible) according to the other standard. Since the use of standards changes over time, this could have affected our results over time. To address this issue, we performed sensitivity analyses for changes in carbapenem non-susceptibility over time: 1) restricted to isolates evaluated according to EUCAST since this is the most commonly used standard in our surveillance data; 2) excluding isolates categorized as “intermediate” to one or more carbapenems and not “resistant” to any carbapenem because for some isolates the classification as “sensitive” or “intermediate” might depend on the standard used.

Resistance against some antibiotics is not routinely tested (e.g. colistin), but only when resistance against other antibiotics is found. Since this can introduce bias into the analysis, we conducted a sensitivity analysis for these antibiotics by restricting the analysis to isolates from laboratories that routinely test ≥90% of all isolates against the respective antibiotic.

## Results

**Fig. 1:**
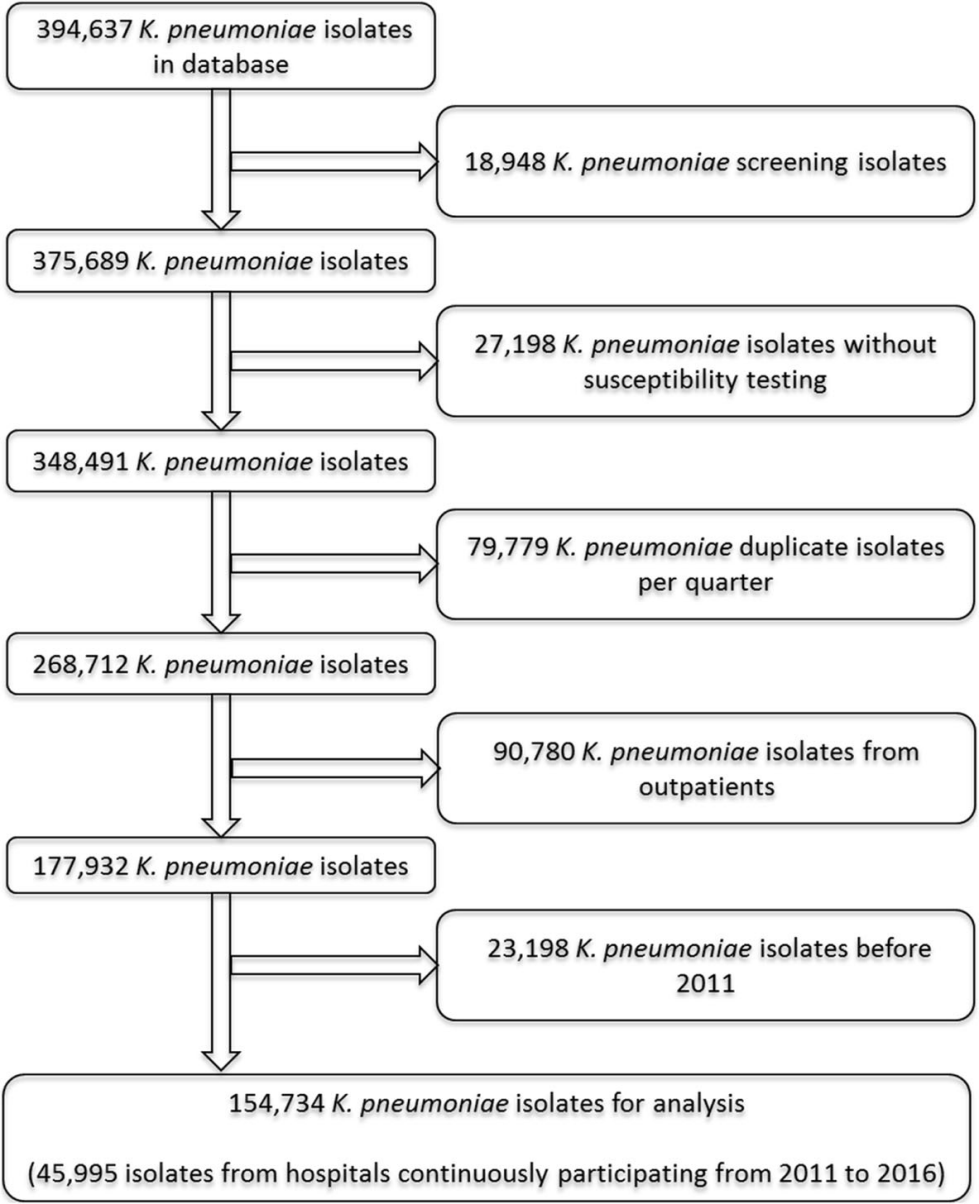
Selection of *K. pneumoniae* isolates from ARS database for the analysis, 2011–2016

Of the 394,637 *K. pneumoniae* isolates in the database we excluded screening isolates, isolates without antimicrobial testing, repeated isolates of the same patient within one quarter, and isolates from outpatients. We included 154,734 isolates between 2011 and 2016 from 655 hospitals in the analysis (Fig. 1).

The median period of data availability per hospital was 2.0 years (IQR: 1.7–4.0 years). Out of all hospitals, 96 had continuous participation in our surveillance system between 2011 and 2016 (45,995 isolates). Hospitals contributing data to the analysis were distributed across Germany with clusters in Schleswig-Holstein, North Rhine-Westphalia, and Baden-Wuerttemberg (Additional file 1: Appendix S2). Over time more hospitals contributed data and the number of isolates strongly increased (Additional file 1: Appendix S3). Interestingly, the number of isolates from continuously participating hospitals almost doubled. Most participating hospitals and included isolates were from the western region in Germany. Further information on the distribution of isolates across subgroups can be found in Additional file 1: Appendices S3 and S4.

Antimicrobial susceptibility testing results were interpreted according to guidelines from the EUCAST for most isolates (76.2%). Other interpretation guidelines used were from the Clinical & Laboratory Standards Institute (CLSI, 15.4%), the German Institute for Standardization (DIN, 7.2%), or no information on interpretation guidelines was given (1.1%). Interpretation of the results according to EUCAST guidelines strongly increased over time (Additional file 1: Appendix S5).

Out of the 154,734 *K. pneumoniae* isolates, 99.9% were tested against at least one carbapenem (Table 1).

**Table 1:** Carbapenem non-susceptibility of *K. pneumoniae* isolates in Germany 2011–2016 (*n* = 154,734)

Tested carbapenem	Number of isolates tested (% of all isolates)	Isolates tested “susceptible” (% with 95% CI)^a^	Isolates tested “intermediate” (% with 95% CI)^a^	Isolates tested “resistant” (% with 95% CI)^a^	Isolates tested “non-susceptible” (% with 95% CI)^a^
Meropenem	150,746 (97.4%)	99.58% (99.49, 99.65%)	0.09% (0.07, 0.12%)	0.33% (0.27, 0.40%)	0.42% (0.35, 0.51%)
Imipenem	150,114 (97.0%)	99.62% (99.54, 99.69%)	0.11% (0.09, 0.14%)	0.27% (0.22, 0.34%)	0.38% (0.31, 0.46%)
Ertapenem	56,560 (36.6%)	98.95% (98.68, 99.16%)	0.11% (0.08, 0.16%)	0.94% (0.74, 1.2%)	1.05% (0.84, 1.32%)
At least one carbapenem^b^	154,524 (99.9%)	99.37% (99.24, 99.49%)	0.11% (0.08, 0.13%)	0.52% (0.42, 0.64%)	0.63% (0.51, 0.76%)

^a^ 95% CI calculated accounting for clustering on hospital level

^b^Meropenem, imipenem or ertapenem

The proportion of isolates non-susceptible against at least one carbapenem was 0.63% (95% CI 0.51–0.76%). In detail, 0.11% (95% CI 0.08–0.13%) of the isolates were tested intermediate against at least one carbapenem and 0.52% (95% CI 0.42–0.64%) were tested resistant. Almost all isolates were tested against meropenem and imipenem, whereas susceptibility against ertapenem was tested in only about a third of the isolates. The proportion of non-susceptible isolates was higher for ertapenem than meropenem or imipenem. A sensitivity analysis restricted to isolates tested against all three carbapenems yielded comparable results (Additional file 1: Appendix S6).

Isolates found non-susceptible to meropenem or imipenem were often non-susceptible against the two other carbapenems as well (Table 2).

**Table 2:** Cross-resistance of *K. pneumoniae* isolates non-susceptible against one carbapenem to other tested carbapenems

	Additional non-susceptibility (R + I) against		
Non-susceptibility (R + I) against	Meropenem	Imipenem	Ertapenem
Meropenem (*n* = 634)	–	501/619 (80.9%)	299/314 (95.2%)
Imipenem (*n* = 569)	501/562 (89.1%)	–	252/272 (92.6%)
Ertapenem (*n* = 596)	252/591 (42.6%)	299/590 (50.7%)	–

*R* resistant, *I* intermediate

Interestingly, only about half of the isolates tested non-susceptible against ertapenem were also found to be non-susceptible against meropenem or imipenem. A sensitivity analysis restricted to isolates that had been tested against all three carbapenems yielded similar results (Additional file 1: Appendix S7). In addition, we found that out of the 501 isolates tested non-susceptible against both imipenem and meropenem, 139 (27.7%) were tested as intermediate against one or both of these substances.

Non-susceptibility of isolates against at least one carbapenem increased from 0.35% in 2011 to 0.78% in 2016 (Table 3).

**Table 3:** Non-susceptibility of *K. pneumoniae* isolates against at least one carbapenem

		Percentage non-susceptible (R + I) against at least one carbapenem^a^ (95%CI)^b^	
Year	Number of hospitals	All hospitals	Continuously participating hospitals (*n* = 96)
2011	180	0.35% (0.18%; 0.69%)	0.30% (0.17%; 0.51%)
2012	306	0.46% (0.31%; 0.68%)	0.27% (0.17%; 0.41%)
2013	363	0.49% (0.36%; 0.67%)	0.28% (0.18%; 0.45%)
2014	266	0.74% (0.53%; 1.04%)	0.78% (0.46%; 1.33%)
2015	372	0.66% (0.54%; 0.80%)	0.56% (0.35%; 0.89%)
2016	418	0.78% (0.58%; 1.06%)	0.53% (0.35%; 0.81%)

*R* resistant, *I* intermediate, *CI* confidence interval

^a^Meropenem, imipenem or ertapenem

^b^95% CI calculated accounting for clustering on hospital level

Since the number of the participating hospitals changes over time, we restricted the analysis to 96 hospitals that continuously contributed data to the analyses between 2011 and 2016. In these hospitals carbapenem non-susceptibility appears to be stable until 2013 and then increased over the following years. We found evidence for an increasing trend of carbapenem non-susceptibility in the continuously participating hospitals (OR = 1.20 per year, 95% CI 1.09–1.33, *p* < 0.001). The sensitivity analyses 1) restricted to isolates evaluated according to EUCAST and 2) excluding isolates labelled as “intermediate” to one or more carbapenems and not “resistant” to any carbapenem yielded similar results (Additional file 1: Appendix S8).

In uni- and multivariable analyses for risk factors associated with carbapenem non-susceptibility, we could observe that isolates collected from men were more likely to be non-susceptible against at least one carbapenem than isolates from women (Table 4 and Additional file 1: Appendix S9).

**Table 4:** Analysis of risk factors associated with carbapenem non-susceptibility of *K. pneumoniae* isolates

	Univariable analysis of risk factors associated with carbapenem non-susceptibility		Multivariable analysis of risk factors associated with carbapenem non-susceptibility	
	OR (95% CI)^a^	*p*-value^b^	OR (95% CI)^c^	*p*-value^b^
Year				
2011	1	–	1	–
2012	1.00 (0.64, 1.57)	0.994	0.99 (0.63, 1.56)	0.964
2013	1.29 (0.84, 1.99)	0.239	1.30 (0.85, 2.01)	0.229
2014	1.78 (1.16, 2.75)	0.009	1.84 (1.19, 2.84)	0.006
2015	1.87 (1.23, 2.86)	0.004	1.99 (1.29, 3.05)	0.002
2016	2.00 (1.32, 3.05)	0.001	2.10 (1.38, 3.22)	0.001
Quarter				
Jan - Mar	1	–	1	–
Apr - Jun	1.14 (0.93, 1.40)	0.217	1.13 (0.92, 1.39)	0.248
Jul - Sept	1.07 (0.87, 1.31)	0.515	1.06 (0.87, 1.30)	0.553
Oct - Dec	1.05 (0.86, 1.30)	0.618	1.07 (0.87, 1.31)	0.542
Age (years)				
< 1	0.49 (0.21, 1.11)	0.089	0.26 (0.11, 0.61)	0.002
1–19	2.28 (1.45, 3.58)	< 0.001	1.67 (1.06, 2.66)	0.029
20–39	2.87 (2.17, 3.79)	< 0.001	2.38 (1.78, 3.18)	< 0.001
40–49	1.88 (1.33, 2.64)	< 0.001	1.49 (1.05, 2.10)	0.025
50–59	2.10 (1.63, 2.70)	< 0.001	1.62 (1.26, 2.10)	< 0.001
60–69	1.73 (1.37, 2.18)	< 0.001	1.35 (1.06, 1.71)	0.013
70–79	1.57 (1.28, 1.94)	< 0.001	1.33 (1.08, 1.65)	0.007
80+	1	–	1	–
Sex				
Male	1.81 (1.57, 2.10)	< 0.001	1.57 (1.35, 1.83)	< 0.001
Female	1	–	1	–
Hospital Care Level				
Secondary Care	1	–	1	–
Tertiary Care	3.52 (2.02, 6.11)	< 0.001	2.68 (1.55, 4.66)	< 0.001
Specialist Care	2.16 (1.39, 3.37)	0.001	2.44 (1.57, 3.78)	< 0.001
Prevention and Rehabilitation Care	1.92 (0.93, 3.98)	0.078	2.22 (1.09, 4.52)	0.028
Other	0.91 (0.26, 3.21)	0.886	1.03 (0.29, 3.61)	0.966
Type of care				
Intensive care unit	2.58 (2.20, 3.03)	< 0.001	2.37 (1.98, 2.84)	< 0.001
Normal hospital ward	1	–	1	–
Other	1.96 (1.26, 3.06)	0.003	1.69 (1.08, 2.64)	0.022
Clinical specialty				
Surgery and related	1.50 (1.24, 1.82)	< 0.001	1.15 (0.94, 1.40)	0.176
Internal medicine	1	–	1	–
Other	1.50 (1.26, 1.78)	< 0.001	0.98 (0.81, 1.18)	0.821
Sample type				
Swabs	2.35 (1.94, 2.85)	< 0.001	1.87 (1.53, 2.29)	< 0.001
Blood culture	1.45 (1.01, 2.06)	0.043	1.08 (0.76, 1.56)	0.661
Puncture	1.46 (0.90, 2.37)	0.126	1.08 (0.66, 1.76)	0.769
Urine	1	–	1	–
Wound	1.90 (1.49, 2.41)	< 0.001	1.45 (1.13, 1.87)	0.003
Respiratory	2.07 (1.69, 2.53)	< 0.001	1.21 (0.97, 1.51)	0.093
Other	3.01 (1.98, 4.58)	< 0.001	2.11 (1.37, 3.23)	< 0.001
Region				
Northwest	1	–	1	–
West	1.70 (0.93, 3.14)	0.087	1.73 (0.98, 3.06)	0.057
Southwest	1.03 (0.55, 1.93)	0.922	1.01 (0.55, 1.84)	0.983
Southeast	1.10 (0.55, 2.20)	0.783	0.89 (0.46, 1.71)	0.721
Northeast	1.57 (0.74, 3.33)	0.241	1.43 (0.69, 2.98)	0.336
County Type				
Rural	1	–	1	–
City	1.34 (0.93, 1.92)	0.119	1.17 (0.74, 1.84)	0.508
Counties by Social Deprivation Index				
1 (lowest deprivation)	1	–	1	–
2	1.29 (0.68, 2.44)	0.444	1.29 (0.70, 2.36)	0.412
3	0.96 (0.51, 1.82)	0.897	0.88 (0.47, 1.64)	0.687
4	0.84 (0.44, 1.61)	0.609	0.79 (0.42, 1.47)	0.452
5 (highest deprivation)	0.99 (0.53, 1.84)	0.971	0.91 (0.49, 1.69)	0.758
Hospital Beds per 10.000 inhabitants				
8.2–57.2	1	–	1	–
57.3–71.5	0.96 (0.59, 1.56)	0.870	0.87 (0.53, 1.42)	0.577
71.6–90.9	0.82 (0.51, 1.33)	0.431	0.62 (0.36, 1.08)	0.093
91.0–219.0	1.49 (0.94, 2.36)	0.087	1.01 (0.58, 1.77)	0.969

Isolates from patients with missing values on the variables were not included in the analysis

*CI* confidence interval, *OR* odds ratio

^a^Hierarchical Logistic Regression model accounting for clustering within counties and hospitals

^b^Wald test

^c^Hierarchical Logistic Regression model accounting for clustering within counties and hospitals adjusting for year, quarter age, sex, hospital care level, type of care, clinical specialty, sample type, region, county type, social deprivation index, and hospital beds per 10,000 inhabitants

Isolates from tertiary care, specialist care, and prevention and rehabilitation care hospitals were more likely to be non-susceptible against at least one carbapenem (Table 4). In addition, isolates from patients in intensive care units were also associated with a greater risk of carbapenem non-susceptibility. Compared to isolates from urine cultures, isolates from swabs, wounds, and other materials also were more likely to be non-susceptible. We found some evidence that isolates from the region “West” had the highest risk of non-susceptibility against at least one carbapenem in multivariable analyses. We found no association of quarter, clinical speciality, county type, social deprivation index, and hospital beds per 10,000 inhabitants with the outcome.

In the multivariable hierarchical logistical regression model accounting for clustering between counties and hospitals, the intraclass cluster coefficient for the variance of carbapenem non-susceptibility between counties was 5.9% and between hospitals 11.8%. Overall, 17.8% of the variance observed was attributable to clustering by county and hospital.

**Fig. 2:**
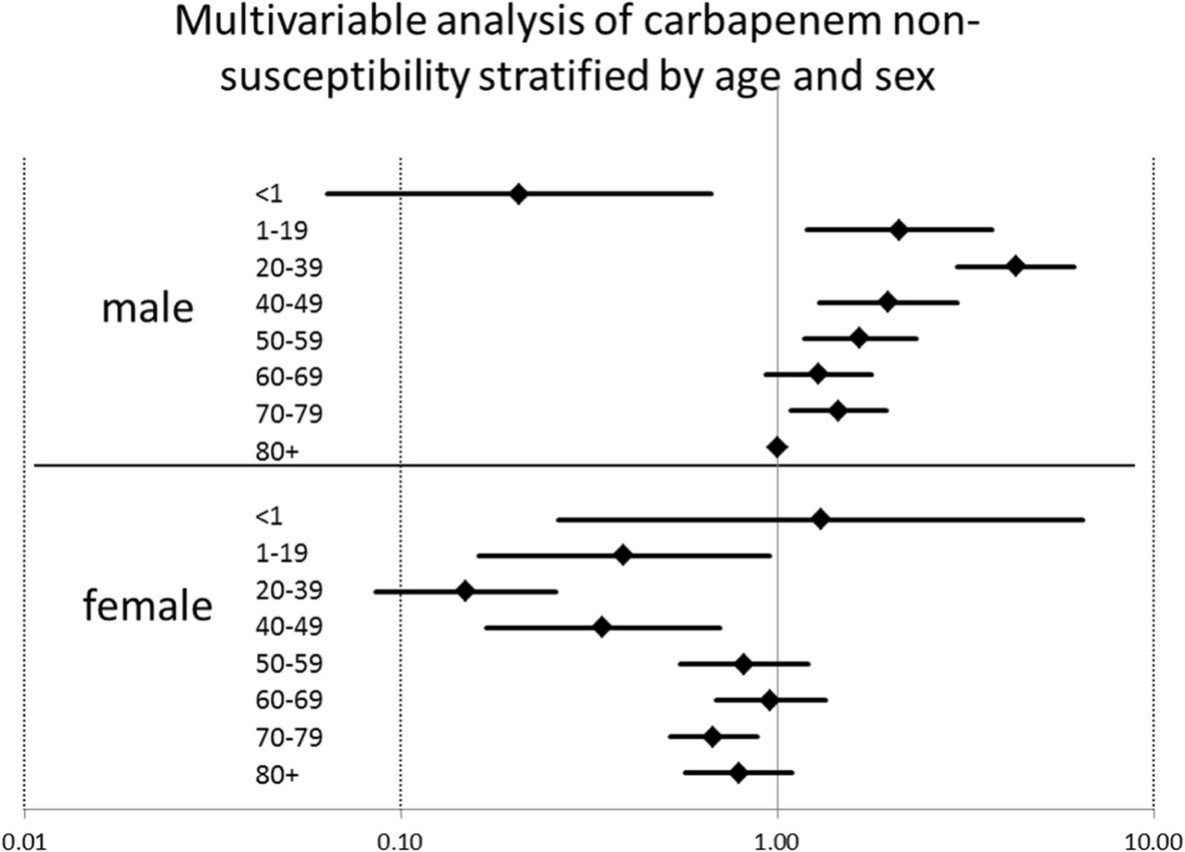
Multivariable analysis for carbapenem non-susceptibility of *K. pneumoniae* isolates stratified by age and sex, Germany 2011–2016. Hierarchical logistic regression accounting for county and hospital, Outcome: carbapenem non-susceptibility stratified by age and sex, adjusted for year, quarter, hospital care level, type of care, clinical speciality, specimen type, region, county type, social deprivation index, and hospital beds per 10,000 inhabitants

When analysed according to age groups, isolates derived from patients 20–39 years had the highest proportion of non-susceptible isolates (1.17, 95%CI 0.79–1.71%) (Additional file 1: Appendix S9). A stratification of carbapenem non-susceptibility across age groups according to sex showed that the increase in non-susceptibility in the younger age groups affected only isolates from men whereas isolates from women had a decreased chance for carbapenem non-susceptibility in both uni- and multivariable analyses (p for interaction < 0.001) (Fig. 2, Additional file 1: Appendix S10). Moreover, this pattern remained unchanged in analyses excluding isolates from gynaecology / obstetric wards or excluding isolates from urine samples (Additional file 1: Appendix S10).

For the 966 isolates non-susceptible to at least one carbapenem, we investigated co-resistance to other antibiotics (Table 5).

**Table 5:** Co-resistance of carbapenem non-susceptible *K. pneumoniae* isolates (*n* = 966) against other antibiotics

	Isolates from all laboratories		Isolates from laboratories that routinely test against the specified antibiotic^a^	
Tested against	Included isolates	Non-susceptible isolates, % (95%CI)^b^	Included isolates	Non-susceptible isolates, % (95%CI)^b^
Ampicillin/ Sulbactam	871	93.0% (90.3, 95.0%)	c	c
Piperacillin/ Tazobactam	950	90.7% (87.4, 93.2%)	c	c
Ceftazidime	954	88.7% (85.8, 91.0%)	c	c
Cefotaxime	908	88.3% (85.0, 91.0%)	c	c
Polymyxin/ Colistin	435	13.3% (9.8, 17.9%)	214	14.0% (8.8, 21.7%)
Tigecycline	865	56.6% (51.0, 62.1%)	709	55.6% (49.1, 61.8%)
Fosfomycin	632	47.0% (40.6, 53.5%)	319	46.7% (36.2, 57.5%)
Gentamicin	960	57.8% (53.0, 62.4%)	c	c
Co-Trimoxazole	961	62.7% (57.6, 67.6%)	c	c
Ciprofloxacin	959	82.0% (78.4, 85.0%)	c	c
Nitrofurantoin	313	82.7% (76.3, 87.7%)	99	72.7% (60.0, 82.6%)

*R* resistant, *I* intermediate, *CI* confidence interval

^a^Analysis restricted to laboratories that test > 90% of isolates against the specified antibiotic

^b^95% CI calculated accounting for clustering by hospital

^c^Antibiotic is routinely tested in all laboratories (> 90% of all isolates tested)

Non-susceptibility against penicillin/beta-lactamase inhibitor combinations, 3rd generation cephalosporins, fluoroquinolones, and nitrofurantoin was high. For tigecycline, fosfomycin, gentamicin, and co-trimoxazole non-susceptibility was found in about half of the isolates. Non-susceptibility was lowest for colistin / polymyxins with 13.3% (95% CI 9.8–17.9%). To investigate selective testing, we restricted the analyses to isolates from laboratories that tested at least 90% of their isolates against the respective antibiotic. The proportion of non-susceptible isolates remained similar to the overall analysis. Out of the 966 isolates non-susceptible to at least one carbapenem, 143 were tested against all antibiotics listed in Table 5 (14.8%). Two of those isolates were tested non-susceptible against all the above mentioned antibiotics, indicating pan-resistant *K. pneumoniae* isolates. In addition, we found 119 isolates that were potentially pan-resistant, i.e. they were tested non-susceptible to the above mentioned antibiotics where investigated, but susceptibility testing results were not available for all these antibiotics.

## Discussion

In this study using routinely collected data on antimicrobial susceptibility testing we found that carbapenem non-susceptibility in *K. pneumoniae* isolates was 0.63% (95% CI 0.51–0.76%) and slowly increasing (OR = 1.20 per year, 95% CI 1.09–1.33, *p* < 0.001). Carbapenem non-susceptibility increased across younger age groups in isolates from men but not from women. Co-resistance against antibiotics of last resort for the treatment of patients with multi-drug resistant bacterial infections was common.

Carbapenem resistance has increased strongly over the last years in some regions, e.g. the United States, China, and southern European countries [5, 17, 18]. In contrast, the proportion of carbapenem non-susceptible isolates in Germany is still low and only slowly increasing. However, we found that the absolute numbers of *K. pneumoniae* isolates from continuously participating hospitals almost doubled from 2011 to 2016, which indicates a growing public health burden concerning *K. pneumoniae* in general and carbapenem non-susceptible *K. pneumoniae* in particular.

The absolute number of *K. pneumoniae* isolates was highest in the older age groups, which is in accordance with the literature [19, 20]. However, upon stratification by age and sex, it is striking that the proportion of carbapenem non-susceptibility is highest in isolates from younger male patients (ages 20–39) and lowest in female patients of the same age groups. Since we do not have further patient-based information available, we can only speculate on the reasons. This effect might be related to differences in patient-based risk factors between men and women at this age. Since we analysed only isolates from hospitals, it is possible that many women in this age group might enter the hospital for child birth while they have a good health status and few risk factors for carbapenem non-susceptibility. Men of the same age group, however, might potentially have more severe reasons for being in the hospital and thus potentially exhibit more risk factors to acquire carbapenem non-susceptible strains. However, sensitivity analyses excluding isolates from potentially healthier patients (from gynaecology / obstetric wards or from urine samples) still showed the same pattern of non-susceptibility and could not explain the observed disparity. Further studies with clinical information are needed to investigate this issue.

Other identified risk factors associated with carbapenem non-susceptibility include isolates from tertiary care, specialist care, and prevention and rehabilitation care hospitals as well as from ICU wards. These findings are in accordance with the literature [20–25] and can be attributed to patients with severe co-morbidities and risk factors for acquiring resistant bacteria. We did not find an association with social deprivation status of the county where the hospital is located and carbapenem non-susceptibility even though social deprivation is associated with poorer health outcomes. It would be interesting to investigate the impact of social deprivation on the patients’ level, which is, however, not available in our surveillance data.

The findings of our study yield valuable information for infection control practices. While measures according to international guidelines including hand hygiene, contact precautions, patient isolation and environmental cleaning are recommended for all health institutions dealing with carbapenem-resistant strains [10], our results highlight that carbapenem non-susceptible isolates were more common in Germany in highly specialized hospitals and intensive care units. Thus, these types of hospitals and hospital units should be especially vigilant and need to implement effective surveillance and infection control measures.

Treatment options for infections with carbapenem non-susceptible *K. pneumoniae* are very limited. While isolates non-susceptible to imipenem or meropenem were mostly non-susceptible to the other two carbapenems as well, in contrast only about half of the isolates non-susceptible to ertapenem showed non-susceptibility to the other two carbapenems. Ertapenem being the more sensitive parameter for carbapenemase producing isolates has been described in the literature [26, 27]. This underlines that Ertapenem non-susceptible isolates should be tested for carbapenemases even when they are still susceptible to meropenem and imipenem. Moreover, in about one third of the isolates tested non-susceptible against imipenem or meropenem we found intermediate susceptibility against one or both substances, which would make them eligible for use in combination therapy. Penicillin/beta-lactamase inhibitor combinations, 3rd generation cephalosporins, fluoroquinolones and nitrofurantoin were not suitable treatment options for most isolates owing to high proportions of non-susceptibility. Less than half of the carbapenem non-susceptible isolates were still susceptible to tigecycline or gentamicin. This is lower than in previous studies and highlights the clinical challenges of infections with carbapenem-resistant *K. pneumoniae* [19, 20, 22, 28]. Non-susceptibility against colistin was observed in 13.3% of the isolates, which is comparable to other studies. However, recent publications question the reliability of routine testing methods for colistin so that the observed result should be interpreted with caution and validated in the future [29, 30]. This is especially important since treatment of carbapenem-resistant *K. pneumoniae* can require combinations of two or three antibiotics often including colistin [28, 31–33]. In this study, we found only two isolates that were non-susceptible to all the antibiotics analysed so that limited treatment options remain for most isolates in this study. However, effective antibiotic stewardship measures should be implemented to keep carbapenem-resistance low and to preserve treatment options [34].

This study uses routine data from accredited laboratories and can base its estimates on isolates from 655 hospitals across the country. This is the first time a study of this size has been conducted in Germany and we are able to investigate even low proportions of carbapenem non-susceptibility in *K. pneumoniae* with satisfactory statistical precision. We included routine data from about one third of all hospitals in Germany [35]. The sample of hospitals is reasonably large and the estimates between a) descriptive analysis and b) regression analysis adjusting for clustering by region and hospital are comparable. Thus, we are confident that our analysis yields valid results and do not expect a major influence of selection bias.

We excluded screening isolates since these have a different probability of showing resistance than clinical isolates. However, since we do not have any clinical patient information, it is possible that some isolates, e.g. from swabs or the respiratory tract, were also derived from screening samples. Due to the study design it was not possible to account for this.

Moreover, laboratories used different guidelines to interpret their AST results (EUCAST, CLSI or DIN), which could lead to differences in qualitative interpretation of the results. Owing to those discrepancies, a small proportion of isolates with values close to the breakpoints might have been classified as “sensitive” according to one standard and “intermediate” (i.e. non-susceptible) according to the other. Since the use of EUCAST increased over time, this could have artificially influenced the trend analyses over time. However, since the sensitivity analyses restricted to EUCAST isolates or excluding isolates evaluated as “intermediate” yielded similar results as the main analysis, we do not expect this effect to have a substantial impact on our study.

## Conclusions

Carbapenem non-susceptibility in *K. pneumoniae* isolates in Germany is still low but slowly increasing. Since the overall number of tested *K. pneumoniae* isolates appears to increase as well, this highlights the growing public health burden of carbapenem resistance in Germany. Continued surveillance, implementation of effective infection prevention and control measures, and sustained antibiotic stewardship efforts are necessary to address these problems.

## Additional file

Additional file 1: **Appendix S1.** List of laboratories that participate in ARS and contributed data to this analysis. **Appendix S2.** Geographical distribution of hospitals contributing data to the analysis. **Appendix S3.** *Klebsiella pneumoniae* isolates by year and location of hospital in Germany, 2011–2016. **Appendix S4.** *Klebsiella pneumoniae* isolates by patient and hospital characteristics in Germany 2011–2016. **Appendix S5.** Numbers of *K. pneumoniae* isolates in Germany 2011–2016 included in the analysis stratified by standards used for interpretation of antimicrobial susceptibility testing. **Appendix S6.** Carbapenem non-susceptibility of *K. pneumoniae* isolates in Germany 2011–2016 that were tested against all three carbapenems. **Appendix S7.** Cross-resistance of *K. pneumoniae* isolates non-susceptible to one carbapenem to other carbapenems restricted to isolates tested against all three carbapenems, Germany 2011–2016. **Appendix S8.** Sensitivity analyses to investigate the trend of carbapenem non-susceptibility over time. **Appendix S9.** Proportions of carbapenem non-susceptibility in *K. pneumoniae* isolates across substrata, Germany 2011–2016. **Appendix S10.** Uni- and multivariable analyses for carbapenem non-susceptibility of *K. pneumoniae* isolates stratified by age and sex, Germany 2011–2016. (DOCX 464 kb)

## Abbreviations

AMR: Antimicrobial Resistance
ARS: Antimicrobial Resistance Surveillance
CI: Confidence Intervals
CLSI: Clinical & Laboratory Standards Institute
DIN: Deutsches Institut für Normung (German Institute for Standardization)
EARS-Net: European Antimicrobial Resistance Surveillance Network
EUCAST: European Committee on Antimicrobial Susceptibility Testing
I: Clinically Intermediate
ICU: Intensive Care Unit
IQR: Interquartile Range
*K. pneumoniae*: *Klebsiella pneumoniae*
MIC: Minimum Inhibitory Concentration
OXA-48: Oxacillinase 48
R: Clinically Resistant
S: Clinically Susceptible

## References

[1] Lee CR, Lee JH, Park KS, Kim YB, Jeong BC, Lee SH (2016). Global dissemination of Carbapenemase-producing Klebsiella pneumoniae: epidemiology, genetic context, treatment options, and detection methods. Front Microbiol.

[2] Gomez-Simmonds A, Uhlemann AC (2017). Clinical implications of genomic adaptation and evolution of Carbapenem-resistant Klebsiella pneumoniae. J Infect Dis.

[3] World Health Organization: WHO publishes list of bacteria for which new antibiotics are urgently needed. http://www.who.int/mediacentre/news/releases/2017/bacteria-antibiotics-needed/en/, accessed on 25.09.2017. 2017.

[4] Correa L, Martino MD, Siqueira I, Pasternak J, Gales AC, Silva CV, Camargo TZ, Scherer PF, Marra AR (2013). A hospital-based matched case-control study to identify clinical outcome and risk factors associated with carbapenem-resistant Klebsiella pneumoniae infection. BMC Infect Dis.

[5] European Centre for Disease Prevention and Control (2017). Surveillance of antimicrobial resistance in Europe 2016. Annual report of the European antimicrobial resistance surveillance network (EARS-net). Stockholm: ECDC.

[6] Ducomble T, Faucheux S, Helbig U, Kaisers UX, Konig B, Knaust A, Lubbert C, Moller I, Rodloff AC, Schweickert B, Eckmanns T (2015). Large hospital outbreak of KPC-2-producing Klebsiella pneumoniae: investigating mortality and the impact of screening for KPC-2 with polymerase chain reaction. J Hosp Infect.

[7] Steinmann J, Kaase M, Gatermann S, Popp W, Steinmann E, Damman M, Paul A, Saner F, Buer J, Rath P. Outbreak due to a Klebsiella pneumoniae strain harbouring KPC-2 and VIM-1 in a German university hospital, July 2010 to January 2011. Euro Surveill. 2011;16(33). 21871227

[8] Pfennigwerth N. Bericht des NRZ für gramnegative Krankenhauserreger. Epid Bull. 2017;25:229-33.

[9] Katchanov J, Asar L, Klupp EM, Both A, Rothe C, Konig C, Rohde H, Kluge S, Maurer FP. Carbapenem-resistant gram-negative pathogens in a German university medical center: prevalence, clinical implications and the role of novel beta-lactam/beta-lactamase inhibitor combinations. PLoS One. 2018;13(4):e0195757. 10.1371/journal.pone.0195757 PMC5896976 29649276

[10] World Health Organization (2017). Guidelines for the prevention and control of carbapenem-resistant Enterobacteriaceae, Acinetobacter baumanii and Pseudomonas aeruginosa in health care facilities. Geneva.

[11] Noll I, Schweickert B, Abu Sin M, Feig M, Claus H, Eckmanns T (2012). Antimicrobial resistance in Germany. Four years of antimicrobial resistance surveillance (ARS). Bundesgesundheitsblatt Gesundheitsforschung Gesundheitsschutz.

[12] INKAR Online - Indikatoren, Karten und Graphiken zur Raum- und Stadtentwicklung in Deutschland und in Europa [www.inkar.de]. Accessed 28 May 2018.

[13] Bundesamt für Kartographie und Geodäsie: Verwaltungskarte Deutschland (Länder, Regierungsbezirke, Kreise) VG250. http://http://www.geodatenzentrum.de/auftrag1/archiv/vektor/vg250_ebenen/2016/. Accessed 28 May 2018.

[14] Kroll LE, Schumann M, Hoebel J, Lampert T (2017). Regional health differences – developing a socioeconomic deprivation index for Germany.

[15] Kroll LS (2017). M; Hoebel, J; Lampert, T: **Regionale Unterschiede in der Gesundheit - Entwicklung eines sozioökonomischen Deprivationsindex für Deutschland**. Journal of Health Monitoring.

[16] Wong GY, Mason WM (1985). The hierarchical logistic regression model for multilevel analysis. J Am Stat Assoc.

[17] Gupta N, Limbago BM, Patel JB, Kallen AJ (2011). Carbapenem-resistant Enterobacteriaceae: epidemiology and prevention. Clin Infect Dis.

[18] Hu FP, Guo Y, Zhu DM, Wang F, Jiang XF, Xu YC, Zhang XJ, Zhang CX, Ji P, Xie Y (2016). Resistance trends among clinical isolates in China reported from CHINET surveillance of bacterial resistance, 2005-2014. Clin Microbiol Infect.

[19] Guh AY, Bulens SN, Mu Y, Jacob JT, Reno J, Scott J, Wilson LE, Vaeth E, Lynfield R, Shaw KM (2015). Epidemiology of Carbapenem-resistant Enterobacteriaceae in 7 US communities, 2012-2013. Jama.

[20] van Duin D, Perez F, Rudin SD, Cober E, Hanrahan J, Ziegler J, Webber R, Fox J, Mason P, Richter SS (2014). Surveillance of carbapenem-resistant Klebsiella pneumoniae: tracking molecular epidemiology and outcomes through a regional network. Antimicrob Agents Chemother.

[21] Giannella M, Trecarichi EM, De Rosa FG, Del Bono V, Bassetti M, Lewis RE, Losito AR, Corcione S, Saffioti C, Bartoletti M (2014). Risk factors for carbapenem-resistant Klebsiella pneumoniae bloodstream infection among rectal carriers: a prospective observational multicentre study. Clin Microbiol Infect.

[22] Lin MY, Lyles-Banks RD, Lolans K, Hines DW, Spear JB, Petrak R, Trick WE, Weinstein RA, Hayden MK (2013). The importance of long-term acute care hospitals in the regional epidemiology of Klebsiella pneumoniae carbapenemase-producing Enterobacteriaceae. Clin Infect Dis.

[23] Ny P, Nieberg P, Wong-Beringer A (2015). Impact of carbapenem resistance on epidemiology and outcomes of nonbacteremic Klebsiella pneumoniae infections. Am J Infect Control.

[24] Schwaber MJ, Klarfeld-Lidji S, Navon-Venezia S, Schwartz D, Leavitt A, Carmeli Y (2008). Predictors of carbapenem-resistant Klebsiella pneumoniae acquisition among hospitalized adults and effect of acquisition on mortality. Antimicrob Agents Chemother.

[25] Xu A, Zheng B, Xu YC, Huang ZG, Zhong NS, Zhuo C (2016). National epidemiology of carbapenem-resistant and extensively drug-resistant gram-negative bacteria isolated from blood samples in China in 2013. Clin Microbiol Infect.

[26] McGettigan SE, Andreacchio K, Edelstein PH (2009). Specificity of ertapenem susceptibility screening for detection of Klebsiella pneumoniae carbapenemases. J Clin Microbiol.

[27] CLSI: **Performance Standards for Antimicrobial Susceptibility Testing**. 27th ed. CLSI supplement M100. Wayne, PA: Clinical and Laboratory Standards Institute; 2017 2017.

[28] Tumbarello M, Trecarichi EM, De Rosa FG, Giannella M, Giacobbe DR, Bassetti M, Losito AR, Bartoletti M, Del Bono V, Corcione S (2015). Infections caused by KPC-producing Klebsiella pneumoniae: differences in therapy and mortality in a multicentre study. J Antimicrob Chemother.

[29] Dafopoulou K, Zarkotou O, Dimitroulia E, Hadjichristodoulou C, Gennimata V, Pournaras S, Tsakris A (2015). Comparative evaluation of Colistin susceptibility testing methods among Carbapenem-nonsusceptible Klebsiella pneumoniae and Acinetobacter baumannii clinical isolates. Antimicrob Agents Chemother.

[30] Matuschek E, Åhman J, Webster C, Kahlmeter G. Evaluation of five commercial MIC methods for colistin antimicrobial susceptibility testing for gram-negative bacteria. ECCMID conference, Vienna 2017. Poster. 2017:P161. https://www.escmid.org/escmid_publications/escmid_elibrary/material/?mid=41167. Accessed 28 May 2018.

[31] Capone A, Giannella M, Fortini D, Giordano A, Meledandri M, Ballardini M, Venditti M, Bordi E, Capozzi D, Balice MP (2013). High rate of colistin resistance among patients with carbapenem-resistant Klebsiella pneumoniae infection accounts for an excess of mortality. Clin Microbiol Infect.

[32] Gonzalez-Padilla M, Torre-Cisneros J, Rivera-Espinar F, Pontes-Moreno A, Lopez-Cerero L, Pascual A, Natera C, Rodriguez M, Salcedo I, Rodriguez-Lopez F (2015). Gentamicin therapy for sepsis due to carbapenem-resistant and colistin-resistant Klebsiella pneumoniae. J Antimicrob Chemother.

[33] Stein C, Makarewicz O, Bohnert JA, Pfeifer Y, Kesselmeier M, Hagel S, Pletz MW (2015). Three dimensional checkerboard synergy analysis of Colistin, Meropenem, Tigecycline against multidrug-resistant clinical Klebsiella pneumonia isolates. PLoS One.

[34] de With K, Allerberger F, Amann S, Apfalter P, Brodt HR, Eckmanns T, Fellhauer M, Geiss HK, Janata O, Krause R (2016). Strategies to enhance rational use of antibiotics in hospital: a guideline by the German Society for Infectious Diseases. Infection.

[35] Federal Statistical Office: https://www.destatis.de/DE/ZahlenFakten/GesellschaftStaat/Gesundheit/Krankenhaeuser/Tabellen/KrankenhaeuserJahreVeraenderung.html. 2013.

