# Supplementary material for: Carbapenem non-susceptibility of Klebsiella pneumoniae isolates in hospitals from 2011 to 2016, data from the German Antimicrobial Resistance Surveillance (ARS)
Source: Antimicrob Resist Infect Control. 2018 Jun 5;7:71. doi: 10.1186/s13756-018-0362-9 (PMC5987571; doi:10.1186/s13756-018-0362-9)
Supplement: Supplementary file 1 — Appendix S1. List of laboratories that participate in ARS and contributed data to this analysis. Appendix S2. Geographical distribution of hospitals contributing data to the analysis. Appendix S3. Klebsiella pneumoniae isolates by year and location of hospital in Germany, 2011–2016. Appendix S4. Klebsiella pneumoniae isolates by patient and hospital characteristics in Germany 2011–2016. Appendix S5. Numbers of K. pneumoniae isolates in Germany 2011–2016 included in the analysis stratified by standards used for interpretation of antimicrobial susceptibility testing. Appendix S6. Carbapenem non-susceptibility of K. pneumoniae isolates in Germany 2011–2016 that were tested against all three carbapenems. Appendix S7. Cross-resistance of K. pneumoniae isolates non-susceptible to one carbapenem to other carbapenems restricted to isolates tested against all three carbapenems, Germany 2011–2016. Appendix S8. Sensitivity analyses to investigate the trend of carbapenem non-susceptibility over time. Appendix S9. Proportions of carbapenem non-susceptibility in K. pneumoniae isolates across substrata, Germany 2011–2016. Appendix S10. Uni- and multivariable analyses for carbapenem non-susceptibility of K. pneumoniae isolates stratified by age and sex, Germany 2011–2016. (DOCX 464 kb) [file 13756_2018_362_MOESM1_ESM.docx]

**Carbapenem non-susceptibility of *Klebsiella pneumoniae* isolates in hospitals from 2011 to 2016, data from the German Antimicrobial Resistance Surveillance (ARS)**

**Additional file 1**

**Appendix S1**: List of laboratories that participate in ARS and contributed data to this analysis

**Appendix S2**: Geographical distribution of hospitals contributing data to the analysis

**Appendix S3**: Klebsiella pneumoniae isolates by year and location of hospital in Germany, 2011-2016

**Appendix S4**: Klebsiella pneumoniae isolates by patient and hospital characteristics in Germany 2011-2016

**Appendix S5**: Numbers of K. pneumoniae isolates in Germany 2011-2016 included in the analysis stratified by standards used for interpretation of antimicrobial susceptibility testing

**Appendix S6**: Carbapenem non-susceptibility of K. pneumoniae isolates in Germany 2011-2016 that were tested against all three carbapenems

**Appendix S7**: Cross-resistance of K. pneumoniae isolates non-susceptible to one carbapenem to other carbapenems restricted to isolates tested against all three carbapenems, Germany 2011-2016

**Appendix S8**: Sensitivity analyses to investigate the trend of carbapenem non-susceptibility over time

**Appendix S9**: Proportions of carbapenem non-susceptibility in K. pneumoniae isolates across substrata, Germany 2011-2016

**Appendix S10**: Uni- and multivariable analyses for carbapenem non-susceptibility of K. pneumoniae isolates stratified by age and sex, Germany 2011-2016

Appendix S1: List of laboratories that participate in ARS and contributed data to this analysis

| Abteilung für Labor- und Hygienemedizin, Zentralklinik Bad Berka GmbH |
| --- |
| aescuLabor Hamburg GmbH |
| amedes MVZ für Laboratoriumsdiagnostik und Mikrobiologie Erfurt GmbH |
| amedes MVZ für Laboratoriumsdiagnostik und Mikrobiologie Halle/Leipzig GmbH, Halle/S. |
| amedes MVZ für Laboratoriumsdiagnostik und Mikrobiologie Jena GmbH, Jena |
| amedes MVZ für Laboratoriumsmedizin und Mikrobiologie GmbH, Fürstenfeldbruck |
| amedes MVZ für Labordiagnostik und Mikrobiologie Rhein-Main GmbH, Raunheim |
| Bioscientia Institut für Medizinische Diagnostik, Labor Ingelheim |
| DRK Krankenhaus Chemnitz-Rabenstein |
| EKA Erzgebirgsklinikum Annaberg gGmbH |
| ELBLAB GmbH Zentrum für LaborMedizin, Meißen |
| Friedrich-Loeffler-Institut für Medizinische Mikrobiologie, Greifswald |
| IMD Labor Oderland, Frankfurt (Oder) |
| Institut für Infektionsmedizin, Universitätsklinikum Schleswig-Holstein, Campus Kiel |
| Institut für Laboratoriumsmedizin, Klinikum Oberlausitzer Bergland, Zittau |
| Institut für Laboratoriumsmedizin, Klinikum Sindelfingen-Böblingen |
| Institut für Laboratoriumsmedizin, Mikrobiologie und Hygiene, Evangelisches Krankenhaus Bielefeld |
| Institut für Laboratoriumsmedizin/Mikrobiologie und Krankenhaushygiene, Lausitzer Seenland Klinikum, Hoyerswerda |
| Institut für Labordiagnostik und Hygiene, Helios Dr. Horst Schmidt Kliniken, Wiesbaden |
| Institut für Medizinische Diagnostik, Greifswald |
| Institut für Medizinische Mikrobiologie und Hygiene, Technische Universität Dresden |
| Institut für Medizinische Mikrobiologie und Hygiene, Universitätsklinikum Heidelberg |
| Institut für Medizinische Mikrobiologie und Hygiene, Universitätsklinikum des Saarlandes, Homburg |
| Institut für Medizinische Mikrobiologie, Universitätsklinikum Magdeburg |
| Institut für Mikrobiologie und Infektionsepidemiologie, Universitätsklinikum Leipzig |
| labopart - Medizinische Laboratorien, Labor Elsterwerda |
| Labor 28 MVZ, Berlin |
| Labor am Hygiene-Institut Gelsenkirchen |
| LABOR CHEMNITZ - Zentrum für Diagnostik am Klinikum Chemnitz |
| Labor Dr. Limbach & Kollegen, Heidelberg |
| Labor Dr. Wisplinghoff, Köln |
| Labor Eveld & Kollegen, Essen |
| LABOR STABER Bayreuth |
| LABOR STABER Dresden – Klipphausen |
| LABOR STABER Nürnberg |
| LADR GmbH Medizinisches Versorgungszentrum Plön |
| LADR MVZ Nord-West, Schüttorf |
| Landesuntersuchungsanstalt für das Gesundheits- und Veterinärwesen (LUA) Sachsen, Dresden |
| Medizinisches Versorgungszentrum SYNLAB Leverkusen GmbH |
| MVZ Clotten - Labor Dr. Haas, Dr. Raif & Kollegen, Freiburg |
| MVZ DIAMEDIS Diagnostische Medizin Sennestadt GmbH, Bielefeld-Sennestadt |
| MVZ Dr. Eberhard & Partner, Dortmund |
| MVZ Dr. Stein + Kollegen, Mönchengladbach |
| MVZ Labor Dr. Fenner & Kollegen, Hamburg |
| MVZ Labor Passau |
| MVZ Medizinisches Labor Münster |
| MVZ wagnerstibbe für Medizinische Mikrobiologie, Infektiologie, Hygiene und Tropenmedizin GmbH, Göttingen |
| SYNLAB Medizinisches Versorgungszentrum Berlin GmbH |
| Zentrallabor Friedrich-Ebert-Krankenhaus Neumünster |
| Zentrallabor Rudolf Virchow Klinikum, Glauchau |
| Zentrum für Klinische Chemie, Mikrobiologie und Transfusionsmedizin, Klinikum St. Georg, Leipzig |

Appendix S2: Geographical distribution of hospitals contributing data to the analysis (n = 645)


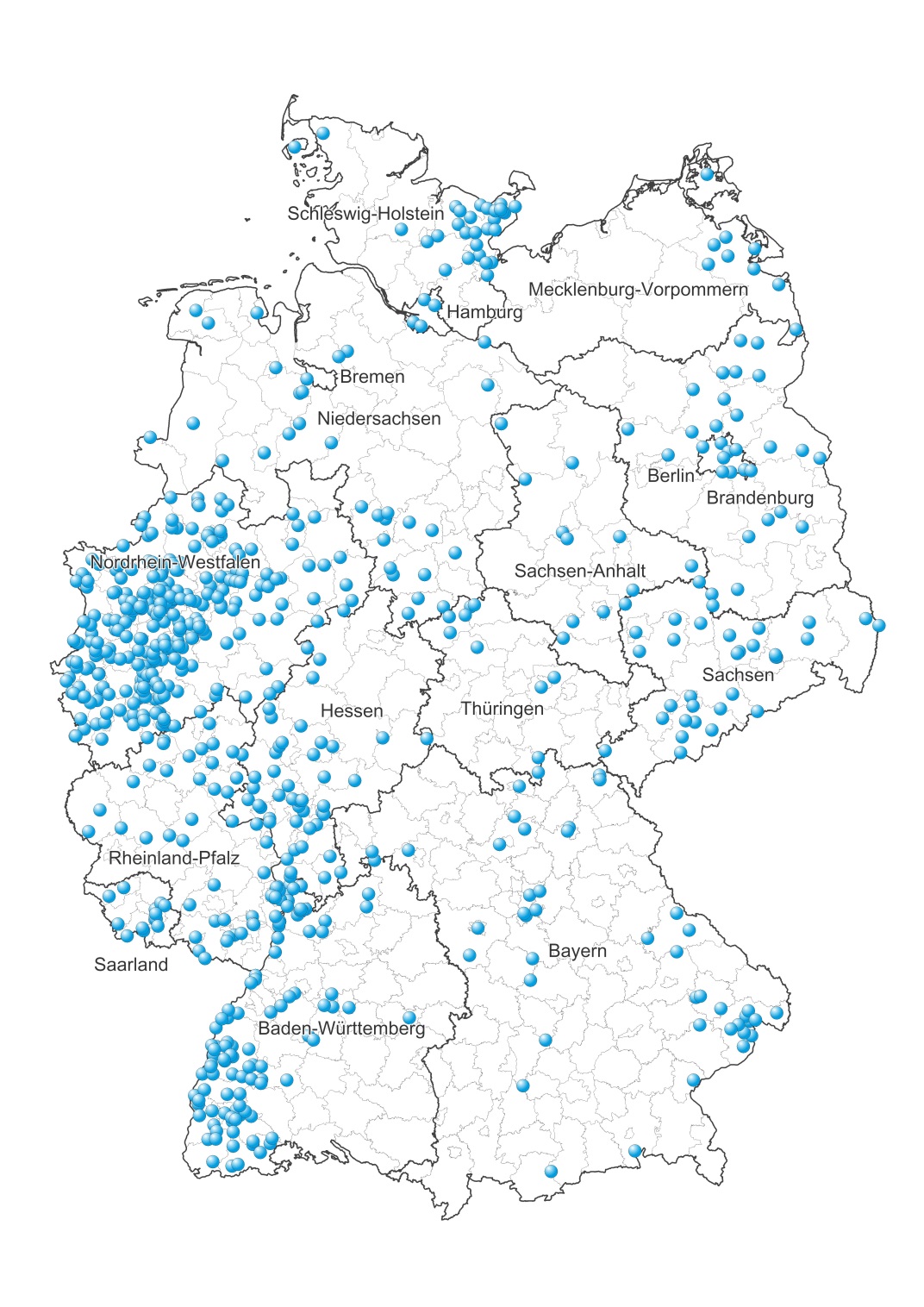


For 10 hospitals contributing 820 isolates to the study no geographical allocation was possible.

Appendix S3: *Klebsiella pneumoniae* isolates by year and location of hospital in Germany, 2011-2016

|  | **Contributing hospitals (n)** | **Isolates (n)** | **Isolates from continuously participating hospitals (n = 96)** |
| --- | --- | --- | --- |
| total | 655 | 154,734 | 45,995 |
| **Year** |  |  |  |
| 2011 | 180 | 11,005 | 5,734 |
| 2012 | 306 | 22,088 | 6,402 |
| 2013 | 363 | 24,821 | 6,759 |
| 2014 | 266 | 21,433 | 8,506 |
| 2015 | 372 | 35,060 | 9,163 |
| 2016 | 418 | 40,327 | 9,431 |
| **Region** |  |  |  |
| Northwest | 66 | 13,177 | 3,886 |
| West | 278 | 68,179 | 22,222 |
| Southwest | 165 | 41,083 | 18,645 |
| Southeast | 87 | 20,052 | 779 |
| Northeast | 49 | 11,423 | - |
| Unknown | 10 | 820 | 463 |
| **County type** |  |  |  |
| Rural | 443 | 72,817 | 21,383 |
| City | 202 | 81,097 | 24,149 |
| Unknown | 10 | 820 | 463 |

Appendix S4: *Klebsiella pneumoniae* isolates by patient and hospital characteristics in Germany 2011-2016

| **Age (years)** | **Isolates (n)** | **Isolates from continuously participating hospitals (n = 96)** |
| --- | --- | --- |
| <1 | 3,123 | 1,010 |
| 1-19 | 2,593 | 916 |
| 20-39 | 7,901 | 2,409 |
| 40-49 | 6,321 | 1,791 |
| 50-59 | 14,413 | 4,142 |
| 60-69 | 23,844 | 6,970 |
| 70-79 | 46,388 | 13,600 |
| 80+ | 50,151 | 15,157 |
| Median (IQR) | 74 (62-82) | 74 (62-82) |
| **Sex** |  |  |
| Male | 56,257 | 17,219 |
| Female | 69,130 | 22,019 |
| Unknown | 29,347 | 6,757 |
| **Hospital Care Level** |  |  |
| Secondary Care | 118,810 | 38,529 |
| Tertiary Care | 22,388 | 2,927 |
| Specialist Care | 8,211 | 3,900 |
| Prevention and Rehabilitation care | 1,982 | 95 |
| Other | 1,110 | 544 |
| Unknown | 2,233 | - |
| **Type of care** |  |  |
| Intensive care unit | 21,773 | 5,812 |
| Normal hospital ward | 129,400 | 39,032 |
| Other | 3,561 | 1,151 |
| **Clinical speciality** |  |  |
| Surgery and related | 25,768 | 6,586 |
| Internal medicine | 84,099 | 23,017 |
| Other | 44,867 | 16,392 |
| **Specimen type** |  |  |
| Swabs | 22,038 | 6,005 |
| Blood culture | 6,945 | 1,905 |
| Puncture | 2,635 | 572 |
| Urine | 88,863 | 27,379 |
| Wound | 13,385 | 3,560 |
| Respiratory | 18,404 | 5,867 |
| Other | 2,248 | 638 |
| Unknown | 216 | 69 |

Appendix S5: Numbers of *K. pneumoniae* isolates in Germany 2011-2016 included in the analysis stratified by standards used for interpretation of antimicrobial susceptibility testing

| CLSI: Clinical & Laboratory Standards Institute, DIN: German Institute for Standardization, EUCAST: European Committee on Antimicrobial Susceptibility Testing |
| --- |

Appendix S6: Carbapenem non-susceptibility of *K. pneumoniae* isolates in Germany 2011-2016 that were tested against all three carbapenems (n= 55,918)

| **Tested antibiotic** | **Proportion of isolates tested susceptible** | **Proportion of isolates tested intermediate** | **Proportion of isolates tested resistant** | **Proportion of isolates tested non-susceptible (R+I)** |
| --- | --- | --- | --- | --- |
| Ertapenem | 98.95% (98.68%, 99.17%) | 0.11% (0.08%, 0.15%) | 0.94% (0.74%, 1.2%) | 1.05% (0.83%, 1.32%) |
| Meropenem | 99.44% (99.29%, 99.56%) | 0.13% (0.09%, 0.18%) | 0.43% (0.32%, 0.57%) | 0.56% (0.44%, 0.71%) |
| Imipenem | 99.52% (99.38%, 99.63%) | 0.15% (0.11%, 0.20%) | 0.33% (0.25%, 0.45%) | 0.48% (0.37%, 0.62%) |

Appendix S7: Cross-resistance of *K. pneumoniae* isolates non-susceptible to one carbapenem to other carbapenems restricted to isolates tested against all three carbapenems (n= 55,918), Germany 2011-2016

|  | **Additional non-susceptibility (R+I) against** | | |
| --- | --- | --- | --- |
| **Non-susceptibility (R+I) against** | **Meropenem** | **Imipenem** | **Ertapenem** |
| Meropenem (n = 313) | - | 241/313 (77.0%) | 298/313 (95.2%) |
| Imipenem (n = 269) | 241/269 (89.6%) | - | 249/269 (92.6%) |
| Ertapenem (n = 586) | 249/586 (42.5%) | 298/586 (50.9%) | - |
| R: resistant, I: intermediate | | | |

Appendix S8: Sensitivity analyses to investigate the trend of carbapenem non-susceptibility over time

1. Analysis restricted to isolates evaluated according to EUCAST:

|  | **Percentage non-susceptible (R+I) against at least one carbapenem^1^ (95%CI)^2^** | |
| --- | --- | --- |
| **Year** | all hospitals | continuously participating hospitals |
| 2011 | 0.42% (0.21%; 0.83%) | 0.31% (0.16%; 0.60%)^3^ |
| 2012 | 0.50% (0.33%; 0.76%) | 0.26% (0.17%; 0.41%) |
| 2013 | 0.49% (0.34%; 0.69%) | 0.26% (0.16%; 0.44%) |
| 2014 | 0.75% (0.54%; 1.05%) | 0.78% (0.46%; 1.33%) |
| 2015 | 0.64% (0.50%; 0.82%) | 0.56% (0.35%; 0.89%) |
| 2016 | 0.85% (0.57%; 1.25%) | 0.55% (0.36%; 0.84%) |
| ^1^ Meropenem, imipenem or ertapenem  ² 95% CI calculated accounting for clustering on hospital level  R: resistant, I: intermediate, CI: confidence interval  ^3^ In 2011 EUCAST was not as commonly used in Germany, so only 65 hospitals contributed data to this analysis | | |

1. Analysis excluding isolates categorized as “intermediate” to one or more carbapenems and not “resistant” to any carbapenem

|  | **Percentage non-susceptible (R) against at least one carbapenem^1^ (95%CI)^2^** | |
| --- | --- | --- |
| **Year** | all hospitals | continuously participating hospitals |
| 2011 | 0.28% (0.14%; 0.55%) | 0.23% (0.12%; 0.41%) |
| 2012 | 0.39% (0.27%; 0.57%) | 0.23% (0.15%; 0.37%) |
| 2013 | 0.41% (0.30%; 0.57%) | 0.24% (0.15%; 0.38%) |
| 2014 | 0.61% (0.42%; 0.90%) | 0.68% (0.39%; 1.20%) |
| 2015 | 0.52% (0.41%; 0.66%) | 0.45% (0.26%; 0.77%) |
| 2016 | 0.67% (0.49%; 0.94%) | 0.44% (0.28%; 0.67%) |
| ^1^ Meropenem, imipenem or ertapenem  ² 95% CI calculated accounting for clustering on hospital level  R: resistant, CI: confidence interval | | |

1. Regression model to investigate trend of carbapenem non-susceptibility in continuously participating hospitals

| Analysis | Estimate for trend of carbapenem non-susceptibility |
| --- | --- |
| All isolates | OR = 1.20 per year, 95% CI 1.09 - 1.33, p < 0.001 |
| Only isolates evaluated according to EUCAST ^1^ | OR = 1.19 per year, 95% CI 1.05 - 1.34, p = 0.005 |
| Without isolates categorized as “intermediate” to one or more carbapenems and not “resistant” to any carbapenem | OR = 1.22 per year, 95% CI 1.09 - 1.36, p = 0.001 |

^1^ excluding 2011 values since only 65 hospitals used EUCAST that year

Appendix S9: Proportions of carbapenem non-susceptibility in *K. pneumoniae* isolates across substrata, Germany 2011-2016

|  | Number of isolates (n) | **Carbapenem non-susceptible isolates^1^**  **% (95% CI)^2^** |
| --- | --- | --- |
| **total** | 154,734 | 0.63% (0.51%; 0.76%) |
| **Year** |  |  |
| 2011 | 11,005 | 0.35% (0.18%; 0.69%) |
| 2012 | 22,088 | 0.46% (0.31%; 0.68%) |
| 2013 | 24,821 | 0.49% (0.36%; 0.67%) |
| 2014 | 21,433 | 0.74% (0.53%; 1.04%) |
| 2015 | 35,060 | 0.66% (0.54%; 0.80%) |
| 2016 | 40,327 | 0.78% (0.58%; 1.06%) |
| **Quarter** |  |  |
| Jan - Mar | 34,985 | 0.55% (0.42%; 0.73%) |
| Apr - Jun | 35,757 | 0.65% (0.52%; 0.80%) |
| Jul - Sept | 43,916 | 0.65% (0.53%; 0.79%) |
| Oct - Dec | 40,076 | 0.64% (0.50%; 0.82%) |
| **Age (years)** |  |  |
| <1 | 3,123 | 0.22% (0.05%; 0.92%) |
| 1-19 | 2,593 | 1.04% (0.57%; 1.90%) |
| 20-39 | 7,901 | 1.17% (0.79%; 1.71%) |
| 40-49 | 6,321 | 0.84% (0.56%; 1.27%) |
| 50-59 | 14,413 | 0.94% (0.74%; 1.19%) |
| 60-69 | 23,844 | 0.77% (0.63%; 0.95%) |
| 70-79 | 46,388 | 0.63% (0.52%; 0.75%) |
| 80+ | 50,151 | 0.88% (0.70%; 1.11%) |
| **Sex** |  |  |
| Male | 56,257 | 0.88% (0.70%; 1.11%) |
| Female | 69,130 | 0.46% (0.36%; 0.58%) |
| Missing | 29,347 | - |
| **Hospital Care Level** |  |  |
| Secondary Care | 118,810 | 0.48% (0.39%; 0.59%) |
| Tertiary Care | 22,388 | 1.25% (0.87%; 1.81%) |
| Specialist Care | 8,211 | 1.06% (0.77%; 1.46%) |
| Prevention and Rehabilitation Care | 1,982 | 0.91% (0.64%; 1.30%) |
| Other | 1,110 | 0.27% (0.10%; 0.72%) |
| Missing | 2,233 | - |
| **Type of care** |  |  |
| Intensive care unit | 21,773 | 1.33% (1.02%; 1.73%) |
| Normal hospital ward | 129,400 | 0.50% (0.41%; 0.61%) |
| Other | 3,561 | 0.83% (0.36%; 1.90%) |
| **Clinical speciality** |  |  |
| Surgery and related | 25,768 | 0.82% (0.59%; 1.13%) |
| Internal medicine | 84,099 | 0.53% (0.45%; 0.63%) |
| Other | 44,867 | 0.69% (0.51%; 0.92%) |
| **Sample type** |  |  |
| Swabs | 22,038 | 1.03% (0.79%; 1.34%) |
| Blood culture | 6,945 | 0.63% (0.42%; 0.96%) |
| Puncture | 2,635 | 0.80% (0.44%; 1.43%) |
| Urine | 88,863 | 0.41% (0.33%; 0.49%) |
| Wound | 13,385 | 0.78% (0.58%; 1.06%) |
| Respiratory | 18,404 | 0.94% (0.75%; 1.18%) |
| Other | 2,248 | 1.25% (0.84%; 1.84%) |
| Missing | 216 | - |
| **Region** |  |  |
| Northwest | 13,177 | 0.35% (0.22%; 0.56%) |
| West | 68,179 | 0.72% (0.52%; 0.98%) |
| Southwest | 41,083 | 0.51% (0.36%; 0.72%) |
| Southeast | 20,052 | 0.67% (0.46%; 0.96%) |
| Northeast | 11,423 | 0.75% (0.45%; 1.25%) |
| Missing | 820 | - |
| **County Type** |  |  |
| Rural | 72,817 | 0.49% (0.40%; 0.59%) |
| City | 81,097 | 0.75% (0.57%; 0.99%) |
| Missing | 820 | - |
| **Counties by Social Deprivation Index** |  |  |
| 1 (lowest deprivation) | 22,009 | 0.60% (0.39%; 0.92%) |
| 2 | 31,003 | 0.68% (0.53%; 0.88%) |
| 3 | 28,123 | 0.69% (0.37%; 1.27%) |
| 4 | 29,000 | 0.56% (0.39%; 0.81%) |
| 5 (highest deprivation) | 43,779 | 0.61% (0.41%; 0.90%) |
| **Hospital Beds per 10,000 inhabitants** |  |  |
| 8.2 - 57.2 | 38,202 | 0.51% (0.40%; 0.65%) |
| 57.3 - 71.5 | 35,270 | 0.73% (0.46%; 1.18%) |
| 71.6 - 90.9 | 41,226 | 0.41% (0.30%; 0.56%) |
| 91.0 - 219.0 | 39,216 | 0.88% (0.64%; 1.20%) |
| ^1^ Isolate non-susceptible against at least meropenem, imipenem or ertapenem  ² 95% CI calculated accounting for clustering by hospital  CI: confidence interval | | |

Appendix S10: Uni- and multivariable analyses for carbapenem non-susceptibility of *K. pneumoniae* isolates stratified by age and sex, Germany 2011-2016

| **Age** | **Isolates with non-susceptibility (R+I) against at least one carbapenem** | | |
| --- | --- | --- | --- |
|  | **total** | **male** | **female** |
| <1 | 7/3,119 (0.22%) | 3/1,390 (0.22%) | 3/1,134 (0.26%) |
| 1-19 | 27/2,588 (1.04%) | 17/957 (1.78%) | 7/1,225 (0.57%) |
| 20-39 | 92/7,893 (1.17%) | 65/2,074 (3.13%) | 17/4,263 (0.40%) |
| 40-49 | 53/6,309 (0.84%) | 34/2,523 (1.35%) | 10/2,547 (0.39%) |
| 50-59 | 135/14,393 (0.94%) | 72/6,438 (1.12%) | 42/5,257 (0.80%) |
| 60-69 | 184/23,814 (0.77%) | 86/10,565 (0.81%) | 62/8,901 (0.70%) |
| 70-79 | 290/46,335 (0.63%) | 148/18,369 (0.81%) | 92/19,138 (0.48%) |
| 80+ | 178/50,073 (0.36%) | 68/13,886 (0.49%) | 83/26,545 (0.31%) |
| R: resistant, I: intermediate | | | |

| **Age** | **Univariable analysis by age and sex^1^** | | | **Multivariable analysis²** | | |
| --- | --- | --- | --- | --- | --- | --- |
|  | **total** | **male** | **female** | **total** | **male** | **female** |
| <1 | 0.43 (0.19, 0.99) | 0.33 (0.10, 1.07) | 1.27 (0.26, 6.33) | 0.26 (0.11, 0.61) | 0.21 (0.06, 0.67) | 1.30 (0.26, 6.49) |
| 1-19 | 2.16 (1.37, 3.39) | 2.75 (1.58, 4.79) | 0.34 (0.14, 0.83) | 1.67 (1.06, 2.66) | 2.11 (1.20, 3.71) | 0.39 (0.16, 0.95) |
| 20-39 | 2.88 (2.18, 3.81) | 5.07 (3.57, 7.22) | 0.14 (0.08, 0.25) | 2.38 (1.78, 3.18) | 4.30 (3.01, 6.14) | 0.15 (0.09, 0.26) |
| 40-49 | 1.74 (1.23, 2.45) | 2.28 (1.50, 3.47) | 0.30 (0.15, 0.62) | 1.49 (1.05, 2.10) | 1.97 (1.29, 3.00) | 0.34 (0.17, 0.70) |
| 50-59 | 1.90 (1.48, 2.45) | 1.92 (1.36, 2.69) | 0.72 (0.49, 1.07) | 1.62 (1.26, 2.10) | 1.66 (1.18, 2.33) | 0.81 (0.55, 1.21) |
| 60-69 | 1.56 (1.24, 1.97) | 1.47 (1.06, 2.03) | 0.86 (0.62, 1.20) | 1.35 (1.06, 1.71) | 1.29 (0.93, 1.78) | 0.96 (0.69, 1.34) |
| 70-79 | 1.46 (1.19, 1.80) | 1.57 (1.17, 2.10) | 0.59 (0.45, 0.77) | 1.33 (1.08, 1.65) | 1.45 (1.09, 1.95) | 0.68 (0.52, 0.89) |
| 80+ | 1 | 1 | 0.67 (0.48, 0.92) | 1 | 1 | 0.79 (0.57, 1.09) |
| ^1^ Univariable analysis: hierarchical logistic regression accounting for county and hospital, Outcome: carbapenem non-susceptibility, stratified by age and sex  ² Multivariable analysis: hierarchical logistic regression accounting for county and hospital, Outcome: carbapenem non-susceptibility stratified by age and sex, adjusted for year, quarter, hospital care level, type of care, clinical specialty, specimen type, region, county type, social deprivation index, and hospital beds per 10,000 inhabitants | | | | | | |

| **Age** | **Multivariable analysis excluding isolates from gynaecology and obstetric wards^1^** | | | **Multivariable analysis excluding isolates from urine samples^1^** | | |
| --- | --- | --- | --- | --- | --- | --- |
|  | **total** | **male** | **female** | **total** | **male** | **female** |
| <1 | 0.25 (0.11, 0.57) | 0.20 (0.06, 0.64) | 1.31 (0.26, 6.53) | ² | ² | ² |
| 1-19 | 1.54 (0.95, 2.49) | 2.01 (1.14, 3.55) | 0.37 (0.14, 0.94) | 2.04 (1.18, 3.50) | 2.96 (1.59, 5.50) | 0.23 (0.07, 0.79) |
| 20-39 | 3.19 (2.39, 4.27) | 4.37 (3.06, 6.24) | 0.28 (0.15, 0.50) | 2.60 (1.80, 3.75) | 4.94 (3.19, 7.66) | 0.12 (0.06, 0.23) |
| 40-49 | 1.51 (1.07, 2.15) | 1.96 (1.29, 2.99) | 0.35 (0.17, 0.73) | 1.42 (0.91, 2.22) | 2.00 (1.19, 3.38) | 0.24 (0.08, 0.71) |
| 50-59 | 1.66 (1.28, 2.15) | 1.65 (1.17, 2.33) | 0.85 (0.58, 1.27) | 1.73 (1.24, 2.42) | 1.79 (1.16, 2.75) | 0.92 (0.57, 1.48) |
| 60-69 | 1.34 (1.06, 1.70) | 1.28 (0.93, 1.77) | 0.94 (0.67, 1.32) | 1.43 (1.05, 1.97) | 1.32 (0.87, 2.00) | 1.27 (0.85, 1.90) |
| 70-79 | 1.36 (1.10, 1.68) | 1.45 (1.09, 1.95) | 0.70 (0.53, 0.91) | 1.41 (1.05, 1.89) | 1.52 (1.03, 2.23) | 0.81 (0.57, 1.16) |
| 80+ | 1 | 1 | 0.79 (0.57, 1.09) | 1 | 1 | 0.93 (0.57, 1.52) |
| ^1^ Multivariable analysis: hierarchical logistic regression accounting for county and hospital, Outcome: carbapenem non-susceptibility stratified by age and sex, adjusted for year, quarter, hospital care level, type of care, clinical specialty, specimen type, region, county type, social deprivation index, and hospital beds per 10,000 inhabitants  ² Isolates from patients <1 year were excluded owing to data sparsity | | | | | | |
